# Protective neutralizing epitopes in SARS‐CoV‐2

**DOI:** 10.1111/imr.13084

**Published:** 2022-05-22

**Authors:** Hejun Liu, Ian A. Wilson

**Affiliations:** ^1^ Department of Integrative Structural and Computational Biology The Scripps Research Institute La Jolla California USA; ^2^ The Skaggs Institute for Chemical Biology The Scripps Research Institute La Jolla California USA

**Keywords:** antibody, coronavirus, epitope, SARS‐CoV‐2, vaccine, variants

## Abstract

The COVID‐19 pandemic has caused an unprecedented health crisis and economic burden worldwide. Its etiological agent SARS‐CoV‐2, a new virus in the coronavirus family, has infected hundreds of millions of people worldwide. SARS‐CoV‐2 has evolved over the past 2 years to increase its transmissibility as well as to evade the immunity established by previous infection and vaccination. Nevertheless, strong immune responses can be elicited by viral infection and vaccination, which have proved to be protective against the emergence of variants, particularly with respect to hospitalization or severe disease. Here, we review our current understanding of how the virus enters the host cell and how our immune system is able to defend against cell entry and infection. Neutralizing antibodies are a major component of our immune defense and have been extensively studied for SARS‐CoV‐2 and its variants. Structures of these neutralizing antibodies have provided valuable insights into epitopes that are protective against the original ancestral virus and the variants that have emerged. The molecular characterization of neutralizing epitopes as well as epitope conservation and resistance are important for design of next‐generation vaccines and antibody therapeutics.

## 1 INTRODUCTION

The ongoing pandemic coronavirus disease 2019 (COVID‐19) has caused over 400 million infections and 6 million deaths since the first identification of the etiological agent, severe acute respiratory syndrome coronavirus 2 (SARS‐CoV‐2) in late 2019.^1^, ^2^ COVID‐19 is a communicable disease, where the most common symptoms are fever, cough, and shortness of breath.^3^, ^4^ A number of complications, such as pneumonia, acute respiratory distress syndrome, sepsis, and cardiac injury, can lead to severe illness and death. The disease spreads mainly via droplet and aerosol transmission and also through direct or indirect contact with respiratory secretions.^3^, ^5^, ^6^, ^7^, ^8^ Hence, social distancing, masking, and frequent hand washing reduce the opportunity for viral transmission. SARS‐CoV‐2 is a member of the betacoronaviruses in the coronavirus family. Its relatives, such as SARS‐CoV‐1 and MERS‐CoV, were responsible for two human epidemics: severe acute respiratory syndrome (SARS) in 2003 and Middle East respiratory syndrome (MERS) in 2012. Overall, these viruses are highly transmissible with fatality rates ranging from 1%‐35%. Here we assess the immune response to SARS‐CoV‐2, focusing mainly on the antibody response, and how the already impressive and constantly growing database of information on antibody isolation, characterization, and epitope identification can be used to guide design of next‐generation vaccines and antibody therapeutics not only to SARS‐CoV‐2 but to coronaviruses in general.

## 2 BRIEF MOLECULAR VIROLOGY

SARS‐CoV‐2 is an enveloped RNA virus with a mainly spherical, crown‐shaped morphology of about 104 nm in diameter on average (around 92 nm if produced in Vero cells^9^, ^10^).^11^ Like many other coronaviruses, the SARS‐CoV‐2 virion contains single‐stranded positive RNA as its genome wrapped around viral nucleocapsid (N) protein. Its membrane is derived from the host cell in which the viral membrane (M), small envelop (E) and spike (S) proteins are embedded. Its genomic RNA encodes another 16 non‐structural proteins and several other regulatory proteins. Once the virus enters a receptive host cell, its viral RNA undergoes transcription and translation to produce the viral proteins required for both host immune evasion and self‐replication.^3^, ^12^ At the late stage during viral assembly in the host cell, the membrane, envelope, and spike proteins encoded by the virus genomic RNA are translated and assembled to allow virion budding from the cell. The nascent virions can then infect other cells or be transmitted to others in the population. Some recent reviews provide detailed information about the life cycle and molecular virology of the virus.^3^, ^12^, ^13^

## 3 HOST RECEPTOR ACE2 AND CELL TROPISM

The virus can infect alveolar airway epithelial cells, vascular endothelial cells, alveolar macrophages, intestinal epithelial cells, lung type II pneumocytes, ileal absorptive enterocytes, and many other types of cells.^3^, ^14^, ^15^ The cell tropism of the virus is largely determined by the surface spike protein, which binds the host proteinaceous receptor angiotensin converting enzyme 2 (ACE2)^2^, ^3^, ^14^, ^15^, ^16^ and several host attachment co‐factors such as C‐type lectins (DC‐SIGN, L‐SIGN, etc.),^17^, ^18^ heparan sulfate,^19^, ^20^, ^21^, ^22^ and Neuropilin‐1.^23^, ^24^ ACE2 is a zinc carboxypeptidase regulating blood pressure in the renal‐angiotensin system.^25^, ^26^, ^27^, ^28^ It is responsible for conversion of angiotensin II to angiotensin 1‐7,^29^, ^30^ as well as angiotensin I to angiotensin 1‐9.^29^, ^31^ This human enzyme is used as a receptor by several different coronaviruses. It was first identified to be the host receptor for SARS‐CoV‐1,^32^, ^33^, ^34^, ^35^, ^36^, ^37^, ^38^, ^39^, ^40^ and soon after for the common cold coronavirus NL63, a seasonal coronavirus.^41^, ^42^, ^43^, ^44^, ^45^ ACE2 is also the host receptor for SARS‐CoV‐2, consistent with the sequence similarity between the receptor binding domains (RBDs) of the spike proteins of SARS‐CoV‐1 and SARS‐CoV‐2.^1^, ^2^, ^16^, ^46^, ^47^, ^48^, ^49^, ^50^, ^51^ However, the RBD of SARS‐CoV‐2 binds ACE2 with substantially higher affinity compared to other coronaviruses,^52^, ^53^ which may contribute to its high infectivity and transmissibility. Since SARS‐CoV‐2/1 and NL63 bind to the N‐terminal peptidase domain on ACE2,^41^, ^54^, ^55^, ^56^, ^57^ drugs targeting ACE2 may potentially inhibit all three coronaviruses. Engineered ACE2 decoy molecules and antibody 3E8 targeting ACE2 have also been explored.^58^, ^59^, ^60^, ^61^, ^62^, ^63^, ^64^, ^65^, ^66^, ^67^, ^68^, ^69^ However, further work is needed to improve the efficacy of these treatments and to address safety concern regarding the critical role of ACE2 in regulating blood pressure,^25^, ^26^, ^27^, ^28^ interferon signaling,^14^ and vasopressin interaction.^70^

## 4 SPIKE PROTEIN AND VIRAL ENTRY MECHANISM

**FIGURE 1:**
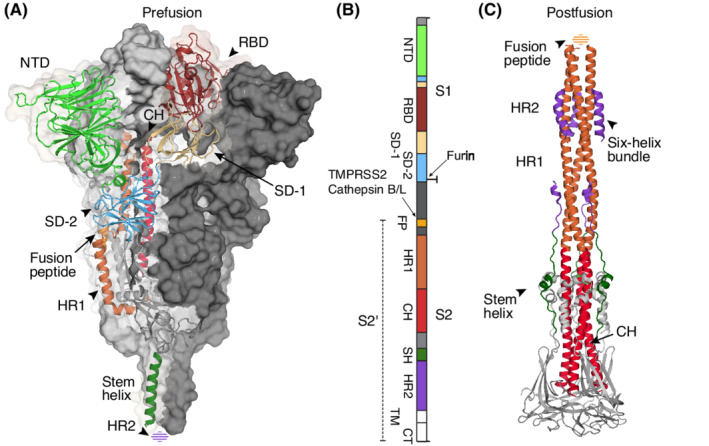
The SARS‐CoV‐2 spike prefusion and postfusion structures. The SARS‐CoV‐2 spike is a trimeric glycoprotein on the surface of the virus. The same colors are used for each domain and subdomains across the panels. (A) The prefusion spike is shown in a dual representation mode. Two of the protomers are shown with their molecular surface (dark and light grey), while the other is shown in a ribbon representation. (B) Domain diagram of full‐length spike. Protease cleavage sites are indicated by arrows. (C) The postfusion spike is shown as a ribbon. Domains within the protomer are colored separately. The spike is first cleaved by a proprotein convertase, such as furin, during biogenesis into two subunits, S1 and S2, that are non‐covalently bound to each other. A secondary cleavage at the S2’ site by TMPRSS2 or cathepsin B/L liberates the fusion peptide (FP) sequence for membrane insertion. Glycans on the spike surface are not shown for simplicity. NTD, N‐terminal domain; RBD, receptor binding domain; SD‐1 and SD‐2, two subdomains in S1 followed RBD. CH, central helix, forms a long helix with HR1, the heptad repeat region 1, in the postfusion state. HR2, heptad repeat region 2 in the prefusion structure (A) and the fusion peptide in the postfusion structure (B) have not been resolved yet and are shown as dashed spheroids. PDBs 6XR8 and 6XRA were used to represent the prefusion and postfusion structures

**FIGURE 2:**
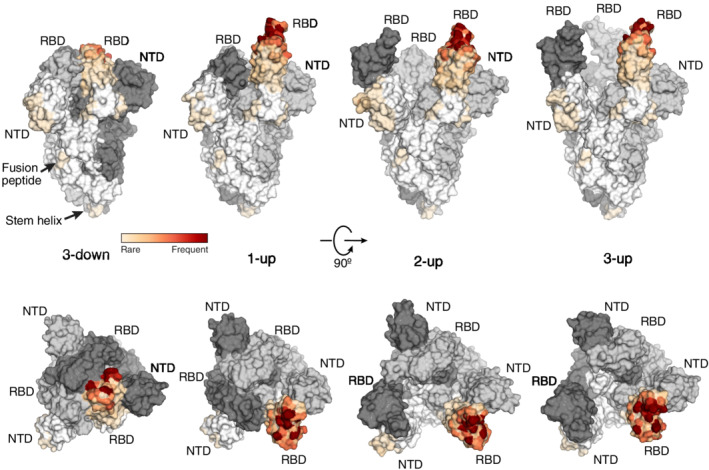
Spike conformations and epitope heatmap of neutralizing antibodies. Spike trimers with different RBD up and down conformations are shown in surface representation (dark grey, light grey, and white for the three protomers of the spike trimer). Epitopes targeted by neutralizing antibodies are mapped onto one protomer. RBD and NTD are the two most vulnerable domains targeted by neutralizing antibodies, while the fusion peptide and stem helix are emerging neutralizing epitope sites targeted by fewer antibodies to date. Neutralizing antibodies with atomic structures in complex with spike or RBD (*n* = 230) were considered for the epitope analysis. The buried surface area (BSA) of each epitope residue by each antibody was calculated using PISA program. Accumulative BSA (aBSA) of each epitope residue is represented on the heatmap. Epitope residues with higher aBSA [from white (low) to red (high)] indicate more vulnerable binding sites for neutralizing antibodies. PDBs 6VXX, 7KJ5, 7CAI, and 7E9O were used to represent spikes with RBS in 3‐down, 1‐up, 2‐up, and 3‐up states, respectively

The virion surface is dominated by the viral spike protein that is responsible for attachment to the host cell surface and for mediating membrane fusion between virus and host cell.^9^, ^10^, ^11^ Unlike most coronaviruses, the spikes of SARS‐CoV‐2, as well as MERS‐CoV, are cleaved by a proprotein convertase, presumably furin, during biogenesis into two non‐covalently linked subunits, S1 and S2 (Figure 1).^52^, ^53^, ^71^, ^72^ The cleaved spike proteins in prefusion and postfusion states, as well as the non‐cleaved form (S0), appear to be present on mature SARS‐CoV‐2 virons.^9^, ^11^, ^73^ Structures of the spike in prefusion and postfusion forms were rapidly determined after SARS‐CoV‐2 was identified.^53^, ^71^, ^74^, ^75^, ^76^, ^77^ S1 consists of an N‐terminal domain (NTD) and receptor binding domain (RBD) followed by two subdomains SD‐1 and SD‐2 (Figure 1B). S2 consists of several regions including the N‐terminal fusion peptide and its proximity region, heptad repeat 1 (HR1), central helix, stem helix, HR2, transmembrane region, and cytoplasmic tail (Figure 1B). The virus binds human receptor ACE2 on the target cell through its RBD on the spike S1. Structural studies have shown that the RBDs in spike can have down, up, and intermediate conformations where the predominant conformations are all down and one up when the RBD is in a native unliganded conformation.^9^, ^10^, ^11^, ^53^, ^65^, ^71^, ^78^, ^79^, ^80^, ^81^ However, the ACE2 receptor binding site (RBS) on the spike is not exposed when the RBD is in a down conformation (Figure 2).^53^, ^71^ As the spike has to expose its RBS to bind ACE2, such exposure can also lead to RBS recognition by antibodies in the immune system.

After engagement with the human receptor, transmembrane serine protease 2 (TMPRSS2) in target cells cleave the spike protein at residue R815,^16^, ^72^, ^82^ leaving a processed S2’ that renders the fusion peptide accessible for membrane fusion with the host target cell (Figure 1).^77^ This process is similar to that observed for SARS‐CoV‐1^83^, ^84^, ^85^ and validated by the TMPRSS2 inhibitor camostat, which inhibits virus infection of TMPRSS2‐positive cells.^16^, ^72^ Precleavage of the SARS‐CoV‐2 spike by proprotein convertase is beneficial to SARS‐CoV‐2 infection of TMPRSS2‐positive cells.^52^, ^72^ Nevertheless, SARS‐CoV‐2 can also infect TMPRSS2‐negative cells. Reagents such as ammonium chloride and hydroxychloroquine that inhibit endosomal acidification can suppress SARS‐CoV‐2 infection in cell‐based assays but not in the clinic.^76^, ^86^ In this case, after engagement by human receptor ACE2 on the target cell surface, the virus through its spike protein is internalized via clathrin‐mediated endocytosis.^73^, ^87^, ^88^ In the endolysosomes, the spike is presumed to be cleaved by proteases cathepsin B/L in a similar way to SARS‐CoV‐1.^16^, ^52^, ^76^, ^86^, ^89^ However, it is not clear whether endocytosis has a major role in SARS‐CoV‐2 pathogenesis, although TMPRSS2 appears to be essential in mouse models of MERS‐CoV and SARS‐CoV‐1 infection.^90^ Recent studies have shown that the mutations in SARS‐CoV‐2 may change the disease severity. The recent Omicron variant (BA.1) replicates faster in upper‐airway bronchi but less efficiently in lung parenchyma or lower‐airway tissues compared to other variants of concern or ancestral strain, which may lead to more dependence on entry through the endocytosis pathway in the upper airway.^73^, ^91^ Another study under review reports that Omicron BA.2 has similar infectivity and pathology in mice and hamsters.^92^

The fusogenic process in respiratory viruses is highly similar and has been widely reviewed for influenza virus,^92^, ^93^, ^94^ HIV,^95^, ^96^ paramyxoviruses,^97^ and coronaviruses including SARS‐CoV‐1, SARS‐CoV‐2, and MERS‐CoV.^98^, ^99^, ^100^ The viral spike is thought to contain a spring‐loaded fusion machinery. In case of SARS‐CoV‐2, binding of ACE2 leads to cleavage at the R815 site, either by TMPRSS2 or cathepsin B/L, and is akin to releasing the safety bolt and liberating the fusion peptide for membrane fusion. The S2’ region then undergoes dramatic structural reorganization to form a super‐long helix that contains HR1 and the central helix (CH) (Figure 1). The fusion peptide is now relocated atop the long helix (approximately 180Å) in the six‐helix bundle in the spike trimer and poised to target the host cell membrane.^77^, ^100^, ^101^ Overall, these concerted conformational changes bring the cell and viral membranes into close proximity that ultimately leads to membrane fusion, which is critical for releasing the viral genome into the target cell. Inhibitors that interrupt the transition from prefusion to postfusion forms prevent infection by several viruses, including influenza virus^102^, ^103^ and SARS‐CoV‐2.^101^, ^104^, ^105^ More details regarding spike proteins of coronaviruses and their cell entry mechanisms can be found in several excellent reviews.^3^, ^18^, ^76^, ^99^, ^106^, ^107^, ^108^, ^109^, ^110^, ^111^

## 5 IMMUNE RESPONSE TO SARS‐COV‐2 INFECTION

SARS‐CoV‐2 infection can lead to strong immune responses.^4^, ^112^ Endosomal toll‐like receptors such as TLR3, TLR7, and TLR8, and cytosolic RIG‐I‐like receptors such as RIG‐I and MDA5 can signal viral invasion and stimulate secretion of type I and III interferons, and nuclear factor κB‐dependent proinflammatory cytokines and chemokines to defend against invasion.^3^, ^4^, ^13^ SARS‐CoV‐2‐specific CD4^+^ and CD8^+^ T cell and B cell responses are also detected in COVID‐19 patients^113^, ^114^, ^115^, ^116^, ^117^ and associated with protective immunity and disease severity.^118^, ^119^, ^120^ Many successful vaccines elicit strong germinal center responses that produce mature B cells such as long‐lived plasma cells and memory B cells, which produce high‐affinity, antigen‐specific antibodies.^121^, ^122^, ^123^, ^124^, ^125^, ^126^ Since the start of the COVID‐19 pandemic, the antibody response has been extensively studied in SARS‐CoV‐2‐infected and vaccinated individuals. A plethora of highly specific antibodies have been isolated, many of which neutralize the virus by blocking viral entry into the host cell. These antibodies bind to specific sites on the spike and either prevent engagement between the viral spike and its receptor ACE2 or inhibit the transition from prefusion to postfusion state. Neutralizing antibody potency is a strong predictor of disease severity and protection from SARS‐CoV‐2 infection and has been widely used to determine the effectiveness and breadth of vaccines against SARS‐CoV‐2 including its variants.^127^, ^128^, ^129^ Nevertheless, antibodies that bind Fc and complement receptors on effector cells may also mediate viral clearance and contribute to the immune protection observed in patients and vaccinated individuals.^130^ Overall, the protective immunity against SARS‐CoV‐2, such as antigen‐specific IgG antibodies and neutralization potency, can last for more than half a year but wane over the time in the majority of COVID‐19 patients and vaccinees, and hence require boosting of the responses by an additional round(s) of vaccination.^127^, ^131^, ^132^, ^133^, ^134^, ^135^, ^136^

## 6 NEUTRALIZING EPITOPES ON SARS‐COV‐2 SPIKE

Currently, tens of thousands of SARS‐CoV‐2 antibodies have been isolated since the start of the pandemic.^137^ However, only a few hundreds have had their structures determined in complex with SARS‐CoV‐2 antigens, such as spike, RBD, NTD, or stem helix and fusion peptides. These antibody‐antigen complex structures enable us to molecularly characterize the neutralizing epitopes and any common features of antibody recognition.

**FIGURE 3:**
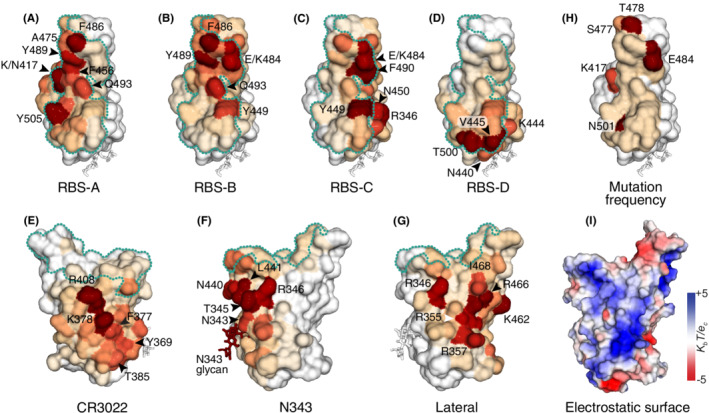
Essential residues in each epitope sites on the RBD. The RBD is shown in surface representation and N343 glycan in stick mode. The footprint of ACE2 binding on the RBS is shown as a cyan dotted line. Epitope sites, RBS‐A (A), RBS‐B (B), RBS‐C (C), RBS‐D (D), CR3022 cryptic site (E), N343 proteoglycan site (F), and lateral RBD site (G) are colored according to aBSA normalized within each epitope group. The most vulnerable epitope residues are indicated in each panel. (H). Mutational frequency (from white to beige to red) in SARS‐CoV‐2 RBD using genomic analysis data from GISAID. The redder colors represent higher mutational frequency in the SARS‐CoV‐2 RBD. (I). Electrostatic surface of SARS‐CoV‐2 RBD. Charge potential was calculated using APBS plugin in PyMol software. The perspective view is the same as G for easy comparison

**FIGURE 4:**
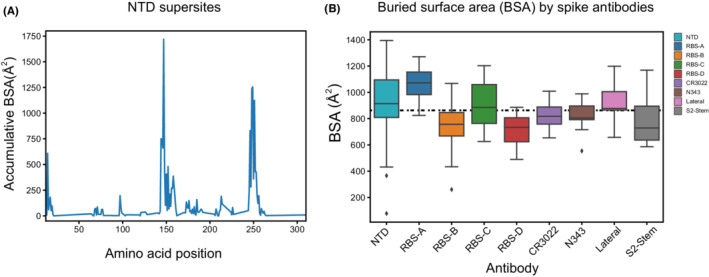
Properties of neutralizing epitopes. A. Two main antigenic supersites in the NTD. Neutralizing antibodies targeting NTD are clustered on two linear regions (D144‐Q158 and E246‐T253). aBSA was calculated from structures of NTD neutralizing antibodies (*n* = 22) using PISA program. B. Average BSA of individual neutralizing antibodies bound to each epitope site. Antibodies targeting RBS‐A and NTD sites are more likely to have larger BSA compared to ACE2 interacting with RBS (dashed line)

Identification of the epitopes, or sites on the antigen where antibodies bind, is critical for understanding how antibody binding can translate into protective immunity established by previous infection and vaccination. The epitopes where neutralizing antibodies bind have been and still are one of the main focus areas for vaccine design and therapeutic antibodies. We and others have reported structures that inform on how neutralizing antibodies recognize the virus and prevent SARS‐CoV‐2 infection. To date, all neutralizing antibodies target epitopes on the spike protein (Figure 2). The most common epitopes are on the RBD and to a lesser extent on the NTD (Figure 2).^138^, ^139^, ^140^, ^141^, ^142^, ^143^, ^144^ Within the RBD, the majority of neutralizing antibodies target or bind close to the RBS. The RBS has a relatively large surface and can be targeted by antibodies that approach the RBS at a variety of angles and interact with different parts of the RBS. These antibodies can be clustered into four major subgroups (RBS‐A to D) based on their epitope preference as we previously proposed (and updated in Figure S1).^145^ Binding sites on the RBD other than the RBS have also been identified. A cryptic epitope site on one of the lateral faces of the RBD was first identified as a binding site for antibody CR3022, a cross‐reactive antibody isolated from a SARS patient.^146^ The N343 proteoglycan site on the opposite lateral face was identified by antibody S309 (Figure 3), a cross‐neutralizing antibody also isolated from a SARS patient.^147^ More recently, another lateral RBD site was identified by antibodies COVOX‐45^148^ and S2H97^148^ (Figure 3). In general, RBS antibodies are usually more potent, while antibodies to the CR3022, N343 proteoglycan, and lateral RBD sites tend to have greater breadth.^149^ However, there are some exceptions of RBS antibodies that have breadth as well as potency, and antibodies to other sites that are potent as well as broad in neutralizing SARS‐CoV‐2. Two linear epitope sites on the NTD (Figure 4A) are often targeted by neutralizing antibodies but less frequently compared to the RBD. When comparing antibodies to these various sites, RBS‐A and NTD antibodies usually have larger antibody–antigen interfaces, that is, buried surface areas (BSAs), versus other RBD antibodies (Figure 4B). Many antibodies also target the S2 subunit but most are not neutralizing, although some show moderate protection in animal models.^150^, ^151^, ^152^, ^153^, ^154^ We will now discuss the characteristics of these epitopes and the propensity for antibodies to target these sites.

### 6.1 RBD epitope sites

RBD is the domain on the spike protein that binds human receptor ACE2. However, the region on the RBD where ACE2 binds (RBS) is not fully accessible when the RBDs are in the down conformation (Figure 2). RBD in the up conformation exposes the receptor binding site and binds ACE2 at nanomolar affinities in the wild‐type and different SARS‐CoV‐2 variants identified so far.^155^, ^156^, ^157^ The virus has retained if not increased its affinity to ACE2 in the emerging variants through mutations within the RBD interface with ACE2; some of these mutations also aid in escape from host immunity. In general, the RBD is highly immunogenic since both SARS‐CoV‐2 infection and vaccination elicit robust antibody responses to the RBD.^123^, ^127^, ^134^, ^158^, ^159^, ^160^, ^161^, ^162^, ^163^, ^164^, ^165^, ^166^, ^167^, ^168^ In many cases, vaccination such as with first‐generation mRNA vaccines elicit higher levels of neutralizing antibodies compared to natural infection.^123^, ^136^, ^158^, ^164^, ^166^ Antibodies targeting the RBS generally compete with ACE2 binding if they bind with high enough affinity. Indeed, many antibody studies have shown that the most potent antibodies target the RBS.^131^, ^139^, ^140^, ^143^, ^145^, ^162^, ^169^, ^170^ Currently, more than 150 neutralizing antibodies with atomic structures have been reported that bind the RBS and block ACE2 binding. The epitopes of these antibodies can be clustered into at least four subgroups.^145^, ^155^ One aim of finely differentiating the epitope sites is to inform on the characteristics and properties of antibodies that bind to each subsite, including germline usage, susceptibility to mutations, where next‐generation vaccines should be targeted, and what is the best combination of antibodies as therapeutics. Antibodies that target different epitope sites have also been combined to reduce the chance of escape mutations during antibody treatment.^171^, ^172^ The purely epitope‐based classification used here is complementary to other classification methods such as by Barnes et al., Denjnirattisal et al., and Cao et al., which are also based on spike conformation (RBD up or down), escape mutations, and antibody competition.^143^, ^169^, ^173^, ^174^

#### 6.1.1 RBS‐A epitope site

Antibodies such as COVA2‐04,^175^ B38,^176^ C102,^169^ CB6,^177^ C105,^75^ BD‐236,^178^ BRII‐196,^160^ C1A‐B12,^179^ NT‐193,^180^ ION‐360^181^ bind similar epitope residues as we originally observed for CC12.1 and CC12.3.^170^ The epitope residues of these antibodies cluster to a specific region roughly corresponding to RBD residues 400‐425, 444‐460, and 473‐506 (except 479‐483), which define an antibody‐targeting subsite in the RBS (Figure S1). This epitope site, designated as RBS‐A,^145^ is largely buried when RBD is in the down state and becomes fully accessible when RBD is in the up conformation.^170^ However, antibodies such as S2H14^182^ and R40‐1G8^183^ can also bind RBS‐A not only when RBD is in the up conformation but also when RBD is in the down conformation if its neighboring RBD is in the up conformation. RBD residues, such as Y505, Q493, F456, K417, Y489, A475, and F486, are essential epitope residues since they contribute extensively to the binding surface and interaction with neutralizing antibodies (Figure 3A). RBD‐A antibodies directly compete with ACE2 binding for neutralization of SARS‐CoV‐2. Moreover, several studies have shown that RBD‐A antibodies, such as BRII‐196, S2H14, and S2K146, can promote S1 shedding and the transition to the postfusion state of the spike in vitro.^184^, ^185^, ^186^ More recently, an ACE2‐mimicking antibody, S2K146, was shown to bind RBS‐A in a similar way to ACE2 but with more than 1000 times higher affinity and trigger the postfusion conformation of the spike trimer.^186^ An Y489H mutation that impairs S2K146 antibody binding also substantially decreases ACE2 binding and leads to a fitness cost compared to wildtype virus.^186^

The RBS‐A antibodies are mainly encoded by the IGHV3‐53 germline gene and the highly related IGHV3‐66 (one amino acid difference) and bind to a concave surface on the RBS using germline‐encoded NY and SGGS motifs in heavy chain complementarity‐determining regions (CDR) H1 and H2.^170^ Little somatic hypermutation seems to be required to achieve strong antibody‐antigen interaction at this site. In fact, many antibodies targeting this site such as CC12.1,^170^ CC12.3,^170^ COVA2‐04,^175^ B38,^176^ C105,^75^ C1A‐B12,^179^ P2B‐1A1,^187^ and S2H14^182^ have almost germline antibody sequences, while others with more somatic hypermutation can have increased breadth and potency.^131^, ^143^ Epitope residues in RBS‐A overlap largely with ACE2 binding residues but interact over much larger interface area than ACE2 (Figure 4B). RBS‐A antibodies often have high neutralization potency against specific SARS‐CoV‐2 strains, such as CC12.3 (IC_50_ 18 ng/mL),^170^ BD‐629 (IC_50_ 6 ng/mL),^170^ BRII‐196 (IC_50_ 30 ng/mL),^184^ and COVOX‐150 (IC_50_ 12 ng/mL)^143^ against the ancestral virus, and β27 (IC_50_ 9 ng/mL) to the Beta VOC.^143^ These types of antibodies can exhibit excellent protection against SARS‐CoV‐2 infection or severe disease in animal models or humans. For instance, CC12.1 (IC_50_ 19 ng/mL) protects mice from SARS‐CoV‐2 infection,^139^ whereas S2K146 (IC_50_ 10 ng/mL) is protective in the Syrian hamster model after intranasal challenge with SARS‐CoV‐2 Beta VOC.^186^

However, many RBS‐A antibodies, as well as other RBS antibodies, are sensitive to mutations found in SARS‐CoV‐2 variants of concern.^149^, ^155^, ^188^, ^189^ Notwithstanding, recent studies have demonstrated that infection by Beta VOC can elicit both strain‐specific and cross‐neutralizing antibodies to RBS‐A, as well as to other RBS sites.^167^, ^168^ Some antibodies isolated from Beta infected patients, such as β22, β27, and β29, potently neutralize Beta and Gamma VOCs, but not the ancestral stain or Alpha VOC.^168^ CS23, isolated from a Beta VOC infected patient in another study, binds specifically to Beta VOC but not the ancestral strain, whereas some others can cross‐neutralize several SARS‐CoV‐2 strains.^167^ The virus now seems to be evolving to escape from immunity established by previous infection and vaccination, but nevertheless is still capable of eliciting new and potent RBS‐A antibodies. Furthermore, RBS‐A antibodies, such as R40‐1G8, COVOX‐222, and S2K146 isolated from early pandemic patients, are both potent and broad in neutralization against a broad spectrum of SARS‐CoV‐2 variant strains including many VOCs.^174^, ^183^, ^186^, ^190^ Other RBS‐A antibodies such as S2H14,^182^ BRII‐196,^191^, ^192^ and NT‐193^180^ are also broad but less potent in neutralizing SARS‐CoV‐2 VOCs or SARS‐CoV‐1. Overall, these findings suggest RBS‐A is capable of eliciting antibodies with both potent and broad protection, although strain‐specific antibodies are more predominant at this epitope site.

#### 6.1.2 RBS‐B epitope site

Structural studies showed that some IGHV3‐53/3‐66 antibodies with long CDRH3 (15 amino acids or longer) or specific somatic hypermutations bind RBS in a distinct conformation compared to those with short CDRH3 or lower somatic hypermutation. These antibodies defined a new epitope site^145^, ^169^, ^175^, ^193^ that we termed RBS‐B.^145^, ^175^ Later, many other germline antibodies were found to target this site and refined the definition of RBS‐B epitope site that mainly covers the RBD ridge (470‐491) and its nearby regions (approximately residues 446‐457 and 492‐505) (Figure S1). The RBS‐B epitope residues have some overlap with both RBS‐A and RBS‐C epitopes as their fairly large footprints encroach to some extent on these adjacent sites. However, the essential epitope residues that interact with neutralizing antibodies are quite distinct (Figure 3B). Residues F486, Y489, E484/K484, Q493, and Y449 generally contribute most to neutralizing antibody binding with F486, Y489, and E484/K484 that are located on the prominent RBD ridge. In almost all of these antibodies, F486 is buried in a pocket at the heavy–light chain interface.^145^, ^193^ Thus, RBS‐B antibodies favor interaction with the RBD ridge. The shape of the RBS‐B surface renders a relatively smaller interface area for antibodies compared to RBS‐A and RBS‐C (Figure 4B). The RBD ridge is also exposed on the surface of the spike regardless of whether RBD is in the up or down conformation. A substantial number of neutralizing antibodies, including C144^169^ and S2M11,^194^ can bind RBD in both up and down states and also interact with the conserved N343 glycan and residues from a neighboring RBD in the spike trimer. Interaction with the neighboring RBD sometimes can lock the spike trimer in a closed RBD down state, which prevents human receptor engagement with the neighboring RBD.^169^, ^194^ Other antibodies, such as COVA2‐39 and S2E12, however, may require additional space for binding RBS‐B and thus can only bind RBD in the up conformation.^169^, ^175^, ^182^

Many neutralizing antibodies targeting RBS‐B are extremely potent against specific SARS‐CoV‐2 strains and show efficacy in protection from SARS‐CoV‐2 infection or severe disease in animal models or humans. For instance, CV07‐209, S2E12, S2M11, CT‐P59, and J08 provide protection in the Syrian hamster model^140^, ^182^, ^195^, ^196^ and AZD7442 in non‐human primates.^197^ REGN10933 and LY‐CoV555 are in the clinic in combination with antibodies targeting other sites, and lowered disease severity when treated in the early stages of COVID‐19 during the initial phases of the pandemic.^198^, ^199^, ^200^, ^201^ Like many other RBS antibodies, RBS‐B antibodies, such as LY‐CoV555, CV05‐163, S‐B8, COVA2‐39, C144, and β26, are susceptible to mutations in SARS‐CoV‐2 variants of concern.^155^, ^168^, ^193^

However, accumulating evidence suggests that the RBS‐B epitope can elicit both potent and broad antibodies against SARS‐CoV‐2 variants. The structure of RBD ridge is retained between SARS‐CoV‐1 and SARS‐CoV‐2, where a disulfide bond between C480 and C488 helps maintain the structural integrity and its conservation. A small patch in the RBD ridge is moderately conserved across SARS‐CoV‐2 strains and even other sarbecoviruses that may account for elicitation of broadly neutralizing antibodies to this region.^202^ Antibodies J08, AZD8895, S2E12, and BRII‐198 broadly neutralize a broad spectrum of SARS‐CoV‐2 variants,^148^, ^192^, ^203^, ^204^ whereas antibodies β47, COVOX‐253, A23‐58.1, and B1‐182.1, also broadly neutralize variants including Omicron VOC (BA.1).^168^, ^205^, ^206^ Some RBS‐B antibodies, such as CS44, and CV07‐287, can neutralize many variants but Omicron only weakly.^167^ Interestingly, all of these antibodies (except BRII‐198) are encoded by a IGHV1‐58 and IGKV3‐20 public clonotype for their heavy and light chain variable regions, respectively. The germline‐encoded paratope residues of these IGHV1‐58 antibodies, such as W50 and Y52 in heavy chain CDR2, and the disulfide bond between C97 and C100b in the heavy chain CDR3 region, favor interaction with the protruding RBD ridge, especially engagement with F486.^167^, ^207^, ^208^ Collectively, the RBS‐B epitope also seems a promising site for therapeutic antibodies and next‐generation vaccine design.

#### 6.1.3 RBS‐C epitope

The RBS‐C epitope is located on the other side on the RBS from RBS‐A and overlaps partially with RBS‐B, N343 proteoglycan, and lateral RBD epitope sites (Figure 3C) RBS‐C contains a region roughly corresponding to residues 340‐360 (except 343 and 350) (Figure S1). Specifically, residues Y449, F490, R346, E484/K484, N450, and R346 are the key residues that interact with neutralizing antibodies to RBS‐C. Since RBS‐C is exposed on the spike surface regardless of RBD conformation, antibodies targeting RBS‐C, such as AZD1061, C104, P36‐5D2, BG1‐24, BG7‐20, and N‐612‐017, can usually bind RBD in both up and down states.^169^, ^173^, ^209^, ^210^ RBS‐C antibodies, such as BG1‐24 and BG7‐20, can bind the RBD in down state while interacting with glycans on NTD and a neighboring “up”‐RBD.^173^ However, some RBS‐C antibodies, such as COVOX‐58, only bind RBD in the down state due to its close proximity to NTD.^204^ Antibodies targeting RBS‐C, such as AZD1061, may synergize with RBS‐B antibodies, such as AZD8895, in neutralization against SARS‐CoV‐2 including Omicron.^204^, ^211^ RBS‐C antibodies can also be very potent, for example 1‐57 (8 ng/mL), β38 (11 ng/mL), S2D106 (7 ng/mL) and BG1‐24 (2 ng/mL).^148^, ^168^, ^173^, ^212^ Notwithstanding that RBS‐C antibodies are also sensitive to mutations in VOCs at E484 and L452, broad neutralization against several variants of concern has been observed in a few antibodies.^204^, ^209^

#### 6.1.4 RBS‐D epitope

Antibodies targeting the RBS‐D site can also block ACE2 binding and prevent viral entry. The RBS‐D epitope is located on the far end of the RBS away from the RBD ridge and consists of both conserved and variable residues across sarbecoviruses. However, a highly conserved patch in RBS‐D is adjacent to and partially overlaps with the highly conserved CR3022 site. Epitope residues V445, T500, K444, and N440 contribute most to binding by RBS‐D neutralizing antibodies (Figure 3D). Unlike other RBS antibodies that are vulnerable to SARS‐CoV‐2 variants, many RBS‐D antibodies, such as REGN10987, ADG‐20, LY‐CoV1404, β40, β55, AZD1061, and AZD8895, can potently and broadly neutralize a broad spectrum of SARS‐CoV‐2 variants, including Alpha, Beta, Gamma, Delta, and to some extent Omicron.^168^, ^197^, ^213^ Currently, structures of neutralizing antibodies targeting RBS‐D are less frequently characterized compared with those targeting RBS‐A and RBS‐B.

#### 6.1.5 Mutations in RBS sites are more frequent than other sites

SARS‐CoV‐2 mutations are largely centered on RBS sites as revealed by analysis of 8,600,000 SARS‐CoV‐2 sequencing data uploaded to GISAID (Figure 3H). So far, the most frequent mutations in these sequences include T478K, N501Y, E484K/Q, K417T/N, and S477N. Combination of these mutations in the context of the different variants retains if not increases the binding affinity between RBD and ACE2.^155^, ^156^, ^157^ We and others have shown that K417N leads to decrease in ACE2 binding, whereas N501Y increases binding affinity between RBD and ACE2.^155^, ^214^, ^215^, ^216^ Thus, K417N is most often accompanied by a concomitant mutation N501Y.^155^, ^215^, ^216^ These mutations are also at sites that represent essential epitope residues for neutralizing antibodies targeting the RBS and their mutation can lead to escape from immunity established by prior infection or vaccination. Nevertheless, several potent RBS antibodies such as S2K146 and S2E12 are highly resistant to mutations in VOCs.^148^, ^186^ Although broad RBS antibodies are much less frequently isolated, they seem to be more abundant in a small fraction of individuals as reported in a recent study.^183^ Moreover, recent studies on antibodies isolated from patients infected by Beta VOC showed that the RBS sites can still elicit both broad and potent antibodies, such as β40 and β55, which neutralize SARS‐CoV‐2 variants including Omicron VOC (BA.1).^167^, ^168^ These findings suggest that the RBS, or at least components of it, can be considered to be important for antibody targeting by vaccines and therapeutics regardless of antigenic drift.

#### 6.1.6 CR3022 cryptic epitope site

At the very start of the pandemic, we reported the structure of a SARS‐CoV‐1 antibody, CR3022, in complex with SARS‐CoV‐2 RBD.^146^ This structure revealed a cryptic antigenic site that is not exposed when the RBD is in the down state on the spike. However, CR3022 does not neutralize SARS‐CoV‐2, but this is likely due to its modest binding affinity (~100 nM) compared to SARS‐CoV‐1 (~1 nM).^146^, ^217^ Several others have also reported antibodies isolated from SARS‐CoV‐1 survivors and COVID‐19 patients that neutralize SARS‐CoV‐1 but poorly neutralize SARS‐CoV‐2 virus.^147^, ^185^, ^218^ Later, we and our collaborators characterized an antibody COVA1‐16 isolated from a 47‐year old COVID‐19 patient that can bind to a similar but not identical epitope site. Unlike CR3022, COVA1‐16 uses a different approach angle that competes with ACE2 binding and effectively neutralize SARS‐CoV‐2, rendering this site to be a neutralizing epitope even though it does not directly overlap with any ACE2 binding residues.^142^, ^219^ The competition of COVA1‐16 with ACE2 in part explains its superior neutralization potency compared to those that do not. Moreover, studies have reported that antibodies targeting CR3022 site, such as S2A4 and S2X259, can induce S1 shedding and premature conversion to the postfusion conformation of the spike protein, which could offer another mechanism of protection.^185^, ^220^

The CR3022 cryptic site is located in the intramolecular interface within a spike trimer. RBD residues K378, R408, F377, Y369, and T385 are the most favored epitope residues targeted by neutralizing antibodies to this site (Figure 3E). Amino acid sequence analysis shows that the CR3022 epitope site is highly conserved across sarbecoviruses, a subgroup of betacoronaviruses including SARS‐CoV‐2 and SARS‐CoV‐1 viruses.^202^ This high sequence similarity indicates functional conservation of this region among these viruses. Many residues at this site are involved in intramolecular interactions among the RBDs within a spike trimer as well as between the S1 and S2 subunits.^219^ For instance, RBD residues R408, K378, K386, and Q414 of one protomer interact with the neighboring RBD of another protomer via polar interactions. S383 and T385 interact with the tops of the spike central helices and their connecting loops to HR1, which undergo dramatic conformational changes in the postfusion structure (Figure 1B‐C).^219^ RBD residues interacting with these S2 regions are thus conserved and may help maintain the prefusion state of the spike until all RBDs are in the up conformation. Mutations in the CR3022 site are less frequent than other sites such as the receptor binding site (Figure 3H). Hence, antibodies targeting this site are more likely to broadly neutralize SARS‐CoV‐2 variants and other related coronaviruses. Other antibodies that target this site, such as ADI‐62113, 2‐36, 10‐40, C022, C118, DH107, REGN10985, S2X35, MW06, and S2X259, neutralize a broad spectrum of SARS‐CoV‐2 variants and other related sarbecoviruses.^148^, ^192^, ^202^, ^221^, ^222^, ^223^, ^224^, ^225^

Although highly conserved residues render CR3022 epitope site an ideal target for broad neutralizing antibodies, relatively few potent antibodies to this site have been isolated. One possibility would be the cryptic nature of this site, which may be less visible to the immune system. The other may be the specific approach angle required to effectively compete with ACE2 binding. All of the most potent neutralizing antibodies observed so far to this site interact with highly similar epitope residues (Figure S1). Thus, relatively high conservation of these residues across SARS‐CoV‐2 variants and other coronaviruses, seems ideal for pan‐sarbecovirus vaccine design, although how to specifically target this site needs to be resolved.

The CR3022 site also seems to naturally favor COVA1‐16‐like antibodies, which have a YYDRxG motif within their CDRH3. Recent studies have shown several broadly neutralizing antibodies, such as ADI‐62113, C022, 10‐40, and 2‐36, bind the CR3022 site in a highly similar way.^192^, ^202^, ^219^, ^221^, ^222^, ^225^ A long CDRH3 containing the YYDRxG motif interacts with essential epitope residues K378, R408, F377, Y369, and T385, some of which are involved in interaction with spike S2 subunit.^219^ We recently reported that the immunoglobulin D gene, *IGHD3‐22*, encodes the YYDRxG motif, and is responsible for the highly similar binding mode used by these antibodies.^202^, ^222^ YYDRxG antibodies have been elicited in both COVID‐19 patients and vaccinees,^202^ albeit at low frequency. Hence, tuning the immune system to elicit such YYDRxG antibodies would be highly beneficial to broad protection against SARS‐CoV‐2 variants and other related viruses.

#### 6.1.7 N343 proteoglycan epitope site

The N343 proteoglycan site is on the opposite face from the CR3022 cryptic site (Figure 3F). It is characterized by N‐glycosylation at residue N343 of SARS‐CoV‐2 RBD. Most residues at this site are more highly conserved compared to the RBS site but less so than the CR3022 site. Unlike the CR3022 site, this site is exposed regardless of whether the RBD is in up, down, or other intermediate states. However, fewer neutralizing antibodies have been isolated to this epitope, possibly due to shielding by the N343 glycan in the center of the epitope. This N‐glycosylation also seems to be important for stability of the RBD. Starr et al. for example reported that mutations at N343 or T345, which remove the glycosylation sequon, lead to decreased expression of the RBD.^214^ We also observed a decrease in protein yield in mutation of the sequon at this site. Protein dynamics simulations have shown that the N343 glycan is important in modulating the dynamics of the RBD conformation.^79^ As this glycan site is highly conserved across different sarbecoviruses, it suggests a vital role for this region of the RBD in viral evolution and function.

Despite the extra barrier to the immune system generated by the N343 glycan, neutralizing antibodies isolated from SARS or COVID‐19 patients have been isolated that target this epitope site. The first neutralizing antibody structurally characterized to target this site was S309, an antibody isolated from a SARS patient.^147^ This antibody later entered human clinical trials and was approved by U.S. Food and Drug Administration (FDA) with emergency use authorization as sotrovimab (Xevudy). This antibody exhibited exceptional breadth against different variants of concern and some other sarbecoviruses with decent potency, although the FDA recently suspended its use due to concerns about its effectiveness against Omicron subvariant BA.2 (https://fda.gov).

We also reported the structure of a human antibody, CV38‐142, isolated from a 70‐year old COVID‐19 patient, that targets this N343 proteoglycan site. CV38‐142 binds the RBD with fewer direct contacts compared to other antibodies targeting this site (Figure S1) but via a plethora of water‐mediated interactions, which in part explains its tolerance to the antigenic differences between SARS‐CoV‐1 and SARS‐CoV‐2.^226^ Several other antibodies isolated from COVID‐19 patients or vaccinees, such as C135, C032, C548, β6, β49, β50, β53, XG014, 47D11, BG10‐19, neutralize SARS‐CoV‐2, and several VOCs,^227^, ^228^ rendering the N343 proteoglycan site as a prime target for broadly neutralizing antibodies.

When ACE2 is bound, glycans on ACE2, such as at N53, would be close to antibodies targeting the N343 site, which could then potentially impact ACE2 binding to the RBD. However, N343 antibodies do not compete with ACE2 binding, at least not strong enough to block ACE2 binding to the RBD.^147^, ^226^ This raises a question of how N343 site is a neutralization epitope. Structure studies of N343 antibodies suggest that potential mechanisms such as cross‐linking of spikes, locking RBD in the down state, antibody‐dependent cytotoxicity and phagocytosis, may facilitate antibody protection against SARS‐CoV‐2 infection.^147^, ^173^, ^226^ Of note, antibodies targeting the N343 proteoglycan and CR3022 sites can act synergistically. COVA1‐16 and CV38‐142 are able to synergize to neutralize SARS‐CoV‐1, SARS‐CoV‐2, and VOCs with enhanced potency and efficacy.^226^ Next‐generation vaccine design should therefore take both N343 proteoglycan and CR3022 sites into consideration since these sites can elicit antibodies that neutralize SARS‐CoV‐2 variants, as well as other sarbecoviruses.

#### 6.1.8 Lateral RBD epitope site

Recently, a lateral RBD site has been shown to be a promising epitope site in eliciting neutralizing antibodies (Figure 3G). The epitope site is close to RBS ridge and overlaps with RBS‐C site. Several lateral RBD antibodies such as COVOX‐45, S2H97, WRAIR‐2057, ION‐300, and N‐612‐056, neutralize SARS‐CoV‐2 and its variants with moderate potency.^143^, ^144^, ^148^, ^181^, ^210^ The epitopes of these antibodies are contained within RBD residues 351‐360 and 457‐473. The residues primarily responsible to antibody binding are R466, K462, R346, R355, and R357, which form two positively charged patches (Figure 3I). Although this site barely overlaps with the RBS, antibody S2H97 can induce premature transition of the spike to the postfusion state, S1 shedding, and low levels of syncytia formation, and thus contribute to the neutralization activity.^148^ Further antibody discovery may identity more potent neutralizing antibodies to this site.

### 6.2 NTD epitope site

NTD is mainly constituted of β‐sheets and connecting loops and positioned proximal to the neighboring RBD in the spike trimer like the petals of a flower (Figure 2). While the exact biological role of the domain remains elusive, several reports suggest that NTD plays a role in binding attachment factors on host cell surface or recruiting heme metabolites to evade antibody immunity.^229^, ^230^ Although NTD is more exposed on the virion surface compared to other components of the S1 subunit, it is highly glycosylated, which probably decreases its overall immunogenicity.^53^, ^71^, ^74^, ^77^, ^231^ Nevertheless, antibodies targeting NTD are frequently isolated and potent in neutralizing specific SARS‐CoV‐2 strains. Antibodies 2‐51 (IC_50_ 7 ng/mL), 2‐17 (IC_50_ 7 ng/mL), 4‐8 (IC_50_ 9 ng/mL), 5‐24 (IC_50_ 8 ng/mL), S2M28 (IC_50_ 11 ng/mL), and COVOX‐159 (IC_50_ 11 ng/mL) are among the most potent NTD antibodies.^143^, ^194^, ^232^ There appears to be a preference for NTD antibodies to be encoded by IGHV1‐24, IGHV3‐33, and IGHV3‐21 germline genes.^168^, ^194^, ^233^, ^234^ Epitope analyses have identified several linear epitope sites in NTD, such as amino acid positions spanning 144‐158 and 246‐253, that contribute to most of the neutralizing antibody recognition. These sites, referred as NTD supersites, are the dominant epitopes within the NTD.^194^, ^232^, ^235^ However, the virus can easily acquire mutations, deletions, and insertions at these sites, which result in fewer neutralizing antibodies with both potency and breadth that target the NTD.^192^, ^194^ Nevertheless, a recent study showed some NTD antibody can neutralize multiple VOC strains with limited breadth,^233^ which suggest NTD epitope sites should remain under consideration.

### 6.3 Neutralizing epitopes in S2 subunit

Many studies have shown that the S2 subunit elicits a substantial portion of SARS‐CoV‐2 specific antibodies.^121^, ^143^, ^183^, ^236^, ^237^, ^238^, ^239^, ^240^ However, most antibodies targeting the S2 subunit are not neutralizing.^150^, ^151^, ^240^ Some of these antibodies may possibly arise from backboosting of a prior antibody response to seasonal coronavirus spike proteins; these antibodies do not directly block viral entry and some may negatively correlate with disease severity.^150^ However, Song et al. reported an S2‐reactive neutralizing antibody, CC40.8, from 36 cross‐reactive sera that showed moderate neutralization.^241^ Li et al. also analyzed 87 S2 antibodies and found one antibody, CV3‐25, that could neutralize SARS‐CoV‐2.^239^ A very recent study isolated several antibodies from SARS‐CoV‐2 donors that binds the fusion peptide region and exhibits neutralization breadth against alpha and beta coronaviruses.^242^ Thus, exploring neutralization epitopes in S2 subunit may be important to target in pan‐coronavirus vaccines since it is highly conserved across betacoronaviruses. Thus, it is essential to continue the search for antibodies to S2 that have not only breadth but potency.

#### 6.3.1 S2 stem helix

One of the main targets so far for neutralizing antibodies to the S2 domain is to a region in S2 spanning residues 1140‐1162. In the prefusion spike, this region forms the S2 stem helix and connects to HR2 region (Figure 1). Both the stem helix and HR2 undergo dramatic conformational changes in going to the postfusion form. Antibodies CC40.8, S2P6, and CV3‐25 were isolated from COVID‐19 patients and bind to the stem helix epitope and may therefore block formation of the postfusion spike.^152^, ^153^, ^243^ This stem helix is highly conserved across human betacoronaviruses, such as SARS‐CoV‐2/1, MERS‐CoV, and seasonal common cold viruses OC43 and HKU1. These antibodies can neutralize SARS‐CoV‐2 with moderate potency as well as other betacoronaviruses since their epitope residues are highly conserved across betacoronaviruses. Pinto et al. showed S2P6 indeed neutralizes SARS‐CoV‐2/1, common cold coronavirus OC43, as well as another sarbecovirus, GD pangolin coronavirus.^152^Moreover, two studies have shown that CC40.8 and S2P6 protect mice and hamsters from SARS‐CoV‐2 challenge.^152^, ^153^ Two recent studies reported that stem helix antibodies could also be isolated from vaccinated COVID‐19 patients and exhibit protection against SARS‐CoV‐2 and MERS‐CoV in animal models, further suggesting universal vaccine design to this stem helix epitope site is promising for eliciting pan‐betacoronavirus protection if potency can be increased.^154^, ^244^ Since the S2 stem helix is highly conserved across betacoronaviruses whether there is a germline convergent response with conserved motifs to betacoronaviruses, warrants further investigation.

#### 6.3.2 Fusion peptide

Antibodies targeting the fusion peptide in S2 that can neutralize viral infection are not uncommon for HIV,^245^, ^246^, ^247^ although this region was not one of the early epitopes to be identified. The fusion peptide in SAR‐CoV‐2 has to be cleaved by either TMPRSS2 or cathepsin B/L to allow membrane fusion between virus and host cell. Antibodies targeting the fusion peptide could block either protease cleavage or insertion of cleaved fusion peptide into host membrane. Interestingly, recent studies have revealed that some antibodies do indeed target the fusion peptide and contribute to SARS‐CoV‐2 neutralization.^242^, ^243^, ^248^ Thus, this fusion peptide region is also a very promising target for pan‐coronavirus vaccine and therapeutic design.

## 7 IMPLICATIONS FOR VACCINE DESIGN

In general, SARS‐CoV‐2 infection and vaccination can elicit a robust immune response and provide protective immunity. We reviewed here the characteristics of over 200 neutralizing human antibodies whose structures have also been determined. A number of neutralizing epitopes have now been discovered on the RBD, NTD, and S2 (stem helix and fusion peptide) of the spike protein. The most desirable antibodies that have both breadth and potency are indeed being discovered, but they have been much more difficult to find, particularly as the SARS‐CoV‐2 virus continues to evolve with greater and greater antigenic variation. Notwithstanding, a few rare antibodies have been isolated that have both breadth and reasonable potency to SARS‐CoV‐2 and variants of concern, including Omicron. The most highly conserved sites in the RBS include a small region of the RBD ridge, the CR3022 site, and N343 proteoglycan site seem to be promising epitope sites for next‐generation vaccine design and therapeutic antibody development. Vaccines that specifically target a combination of these broadly neutralizing epitopes while not eliciting an overabundance of antibodies against the other more variable or non‐neutralizing epitopes will likely be the best strategy against SARS‐CoV‐2 and variants. Given the extraordinary progress over the past two years, it is now possible to consider pan‐coronavirus vaccines and therapeutics with even greater breadth. A number of neutralizing antibodies to the highly conserved S2 domain of the spike have recently demonstrated that regions such as the fusion peptide and stem helix are promising neutralizing epitopes as they are highly conserved in coronaviruses. Thus, it is now possible to capitalize on these advances to pursue pan‐coronavirus vaccines and therapeutics to protect not only from current SARS‐CoV‐2 strains but also from SARS‐CoV‐1 and MERS‐CoV like viruses and other zoonotic coronaviruses with pandemic potential.

## CONFLICT OF INTEREST

None.

## Supporting information


Figure S1

Supplementary Material

## Data Availability

Data sharing is not applicable to this article as no new data were created or analyzed in this study.
