# Supplementary material for: Protective neutralizing epitopes in SARS‐CoV‐2
Source: Immunol Rev. 2022 May 22:10.1111/imr.13084. Online ahead of print. doi: 10.1111/imr.13084 (PMC9348472; doi:10.1111/imr.13084)
Supplement: Supplementary file 1 — Figure S1 [file IMR-9999-0-s001.pdf]

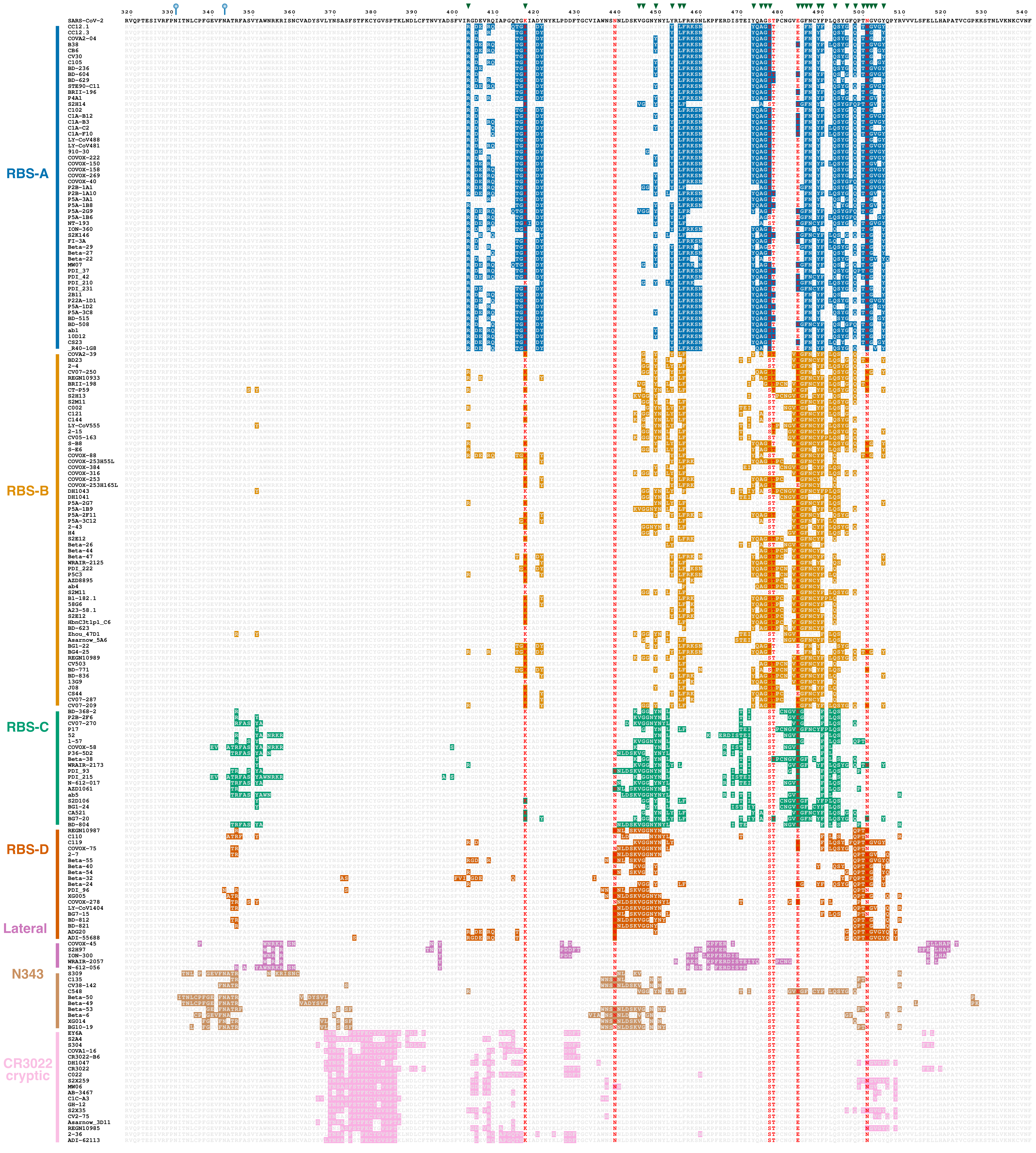

320
330
340
350
360
370
380
390
400
410
420
430
440
450
460
470
480
490
500
510
520
530
540
SARS-CoV-2
RBS-A
RBS-B
RBS-C
RBS-D
Lateral
N343
CR3022 cryptic
