# Supplementary material for: Protective neutralizing epitopes in SARS‐CoV‐2
Source: Immunol Rev. 2022 May 22:10.1111/imr.13084. Online ahead of print. doi: 10.1111/imr.13084 (PMC9348472; doi:10.1111/imr.13084)
Supplement: Supplementary file 2 — Supplementary Material [file IMR-9999-0-s002.docx]

Supplemental Figure 1 **Antibody epitopes aligned to RBD sequence.** Epitopes on the RBD antibodies are mapped onto SARS-CoV-2 RBD sequence. Epitope residues shown in blocks were determined by buried surface area (BSA) >0 Å^2^ as calculated by the PISA program. The most frequent mutations seen in the SARS-CoV-2 GISAID genome data are highlighted in red. Triangles above the sequence table indicate ACE2 interacting residues. Antibodies are grouped by epitope sites in SARS-CoV-2 RBD
